# Estimating the Potential Impact of Canine Distemper Virus on the Amur Tiger Population (*Panthera tigris altaica*) in Russia

**DOI:** 10.1371/journal.pone.0110811

**Published:** 2014-10-29

**Authors:** Martin Gilbert, Dale G. Miquelle, John M. Goodrich, Richard Reeve, Sarah Cleaveland, Louise Matthews, Damien O. Joly

**Affiliations:** 1 Wildlife Conservation Society, Bronx, New York, United States of America; 2 Boyd Orr Centre for Population and Ecosystem Health, Institute of Biodiversity, Animal Health and Comparative Medicine, College of Medical, Veterinary and Life Sciences, University of Glasgow, Glasgow, United Kingdom; 3 Panthera, New York, New York, United States of America; 4 Metabiota, San Francisco, California, United States of America; Mayo Clinic, United States of America

## Abstract

Lethal infections with canine distemper virus (CDV) have recently been diagnosed in Amur tigers (*Panthera tigris altaica*), but long-term implications for the population are unknown. This study evaluates the potential impact of CDV on a key tiger population in Sikhote-Alin Biosphere Zapovednik (SABZ), and assesses how CDV might influence the extinction potential of other tiger populations of varying sizes. An individual-based stochastic, SIRD (susceptible-infected-recovered/dead) model was used to simulate infection through predation of infected domestic dogs, and/or wild carnivores, and direct tiger-to-tiger transmission. CDV prevalence and effective contact based on published and observed data was used to define plausible low- and high-risk infection scenarios. CDV infection increased the 50-year extinction probability of tigers in SABZ by 6.3% to 55.8% compared to a control population, depending on risk scenario. The most significant factors influencing model outcome were virus prevalence in the reservoir population(s) and its effective contact rate with tigers. Adjustment of the mortality rate had a proportional impact, while inclusion of epizootic infection waves had negligible additional impact. Small populations were found to be disproportionately vulnerable to extinction through CDV infection. The 50-year extinction risk in populations consisting of 25 individuals was 1.65 times greater when CDV was present than that of control populations. The effects of density dependence do not protect an endangered population from the impacts of a multi-host pathogen, such as CDV, where they coexist with an abundant reservoir presenting a persistent threat. Awareness of CDV is a critical component of a successful tiger conservation management policy.

## Introduction

Worldwide tiger populations are at an all-time low, with estimated numbers of breeding females reduced to approximately 1,000 animals [1]. Remaining populations are mostly small and fragmented, thus vulnerable to stochastic events that reduce survival of breeding adults [2]. The impact of increased tiger mortality through poaching or human conflict is well known [3], [4], yet the effect of infectious disease outbreaks remains largely unstudied. Recently serological findings suggest that canine distemper virus (CDV) may be an emerging threat to Amur tigers (*Panthera tigris altaica*) in the Russian Far East [5], with clinical cases in 2003 [6], [7] and 2010 [7]. More recently, several cases have been reported in wild tigers in India [8]. The implications of this threat to tiger population dynamics remain unknown.

The morbillivirus causing canine distemper has been recorded in most families of terrestrial carnivores [9]. The virus is capable of causing very high mortality in some species [9], and has been implicated in population declines of African wild dogs (*Lycaon pictus*) [10], Santa Catalina Island foxes (*Urocyon littoralis catalinae*) [11], and black-footed ferrets (*Mustela nigripes*) [12]. Feline CDV infections have been recorded in captive large felids for some time [13], [14]; however, the importance of the virus as a threat to wild felines was not recognized until an outbreak affecting lions (*Panthera leo*) in the Serengeti in 1994 [15], during which an estimated 1,000 animals (approximately 30%) disappeared. Outbreaks have also been recorded in solitary felines with less intra-specific contact than lions, such as such as Iberian lynx, (*Lynx pardinus*) Canadian lynx (*L. canadensis*) and bobcats (*L. rufus*) [16], [17]. Despite the observation of CDV contributing to declines in several endangered populations, many uncertainties remain about the threat that it poses. The Serengeti lion population recovered rapidly following the 1994 epidemic [18], and population viability analyses indicated that the impact of periodic epidemics of CDV on the persistence of Ethiopian wolf (*Canis simensis*) populations was likely to be slight [2]. However, population viability models in African wild dogs suggest that diseases causing high adult mortality can pose a significant extinction threat [19].

Although endangered species are vulnerable to the stochastic effects of infectious disease, pathogens cannot persist in small populations alone, as susceptible hosts are rapidly depleted. However, multi-host pathogens such as CDV can remain a persistent threat where small populations overlap with more abundant susceptible hosts [20]. These species can act individually as reservoir hosts, or collectively as a reservoir community to maintain a pathogen over an extended period. Species within or in contact with the reservoir are then able to act as source populations, capable of transmitting virus to the endangered host [21]. Amur tigers coexist with a range of other carnivores, including three canid, seven mustelid, two ursid and up to three other felid species. Together these represent a diverse community of susceptible and competent CDV hosts, and which along with domestic dogs could act as source populations for tigers, via contact through direct contact such as predatory interaction [22].

This study assesses the threat and impact that CDV poses to the persistence of Amur tigers in a key sub-population in the Russian Far East by means of modelling approaches using various transmission scenarios and population sizes. Moreover, the model is used to determine the differential impact of CDV on isolated populations of varying size, mimicking the actual status of many extant tiger populations.

## Methods

An individual-based stochastic SIRD (susceptible-infected-recovered/dead) model was used to simulate the impact of introducing CDV to the Amur tiger population in Sikhote-Alin Biosphere Zapovednik (SABZ). This site supports a well-studied tiger population with confirmed and suspected cases of CDV, coincident with a period of rapid population decline [7]. The model was developed using the object-oriented programming language Ruby (version 1.8.7), enabling the generation of a virtual tiger population consisting of individual tiger ‘objects’, each with unique attributes (including sex, age and territory occupancy) representing the structure of the wild tiger population. The model updated tiger age in two-week intervals and allowed each tiger to undergo simulated behaviours such as breeding, territory acquisition and death designed to reflect the characteristics of the wild tiger population, using observations and data derived from over 20 years of research on this population. CDV infections were introduced under a range of scenarios to determine the impact on the population growth rate and extinction probability.

### 2.1. Study area and general tiger biology

SABZ (44°46′N, 135°48′E) lies mostly on the eastern slope of the Sikhote-Alin Mountains in Primorskii Krai (province) in the Russian Far East. Access to the Zapovednik (IUCN category I reserve) is strictly limited, but the reserve is surrounded by extensive forests including isolated camps and four small villages. According to 2010 census statistics, 9,800 people live within a 25 km buffer of SABZ, which equates to an estimated 5,444 dogs based on preliminary human: dog density estimates. The Zapovednik and adjacent buffer zone comprise approximately 500 000 ha of suitable tiger habitat, sufficient to support territories for up to 17 female (assuming a home range of 300 km^2^) and five male resident tigers (assuming a home range of 1,200 km^2^). Tiger territories exclude members of the same sex; male territories encompass those of up to five females, with which they defend exclusive breeding rights [23]. Approximately 70% of tiger territories in SABZ extend outside the Zapovednik boundaries, where domestic dogs may be encountered.

### 2.2. Model reproductive parameters

Reproduction in the model was limited to tigers that hold territories. Female tigers become reproductively active at three years of age, giving birth to 1–4 cubs at any time of year [24] (File S1). A probabilistic approach was used to predict litter size for all territorial female tigers over three years old without dependent cubs, using the distribution of litter sizes given in Kerley et al. (2003). Cubs disperse to become non-territory holding ‘floaters’ or inherit vacant territories at approximately 18 months of age [24]. Mean inter-birth interval was 22 months.

### 2.3. Model survival parameters

Age-specific survival was based on estimates derived from radio-telemetry of 42 tigers in SABZ from 1992–2005 [25] and observations of 16 litters of cubs [24]. These estimates were adapted to reflect annual survival in the model (File S1). Telemetry estimates included a period from 1997–2000 when poaching pressure was particularly high, which combined with productivity estimates resulted in a distinctly negative population growth rate (λ = 0.976). As tiger population growth rate is most sensitive to changes in adult survival [26], this parameter was increased proportionally for both sexes from three years of age by adjusting life history traits within a Lefkovitch matrix [27] to produce a population that was approximately stable (λ = 1.0).

### 2.4. CDV infection

An SIRD model was selected to describe CDV infection, as the virus induces life-long immunity in recovered animals [28], and was appropriate to the study objective of assessing outcomes of CDV infection on population growth and persistence. The model assumed that CDV infections arose through direct contact with an infected host and that transmission occurred whenever such contacts took place. The number of susceptible tigers becoming infected in any time step depends on the probability of a susceptible tiger acquiring infection as follows:
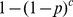
where c is the number of effective contacts per time step (i.e. those where transmission occurs should the contact be with an infectious individual), and p is the prevalence of CDV shedding among those contacts.

Precise measures of CDV-induced mortality in tigers do not exist. We relied on estimates made with reference to published and unpublished case reports (File S1). The most detailed information available was collected during an outbreak in captive tigers at a Texas centre in 2013 when 16 of 22 tigers were clinically infected with CDV; seven died or were euthanized (V. Keahey, personal communication, 2013). The infection status among the six clinically normal animals is unknown, and so mortality rate was approximated as 40% of tigers displaying clinical signs. Data from other published outbreaks was often incomplete or involved small sample sizes. To account for this uncertainty, we also performed sensitivity analyses that set mortality rate to 30%, 40% and 50% to determine how this impacted the outcome. It was assumed that there were no subclinical shedders, and tigers that survived infection acquired life-long immunity to further infection.

The duration of the infectious period of CDV is highly variable, depending on factors including the susceptibility of host species, immune competence and virus strain [29]. Clinical disease has been recorded in captive tigers for periods of a few days to 18 months [6], [13], [14], [30] (File S1); most cases last one to two months. Our model was run with infectious periods of 30, 45 and 60 days. The mortality rate was adjusted for the length of infectious period to ensure it remained at 40% per infection.

### 2.5. Dog to tiger infection

Direct CDV transmission from dogs to tigers was assumed to occur during predation events only. It was assumed that dogs were only predated by the 70% of territorial tigers with ranges extending beyond the Zapovednik boundary, as well as widely ranging non-territorial tigers. Two data sources were used to predict the rate at which these tigers predated dogs (File S1). The first used reported dog predation events in SABZ from 1983–93 [31]. Predation rates were determined from the number of dogs reported killed annually divided by the number of tigers in SABZ at that time [32]. The mean rate of predation per tiger was then taken across all years. This mean of 0.27 dogs/tiger/year is likely to be an underestimate due to under-reporting of dog predation. The second source of predation data was derived from radiotelemetry studies of four tigers [33]. A mean of 1.66 dogs/tiger/year was obtained by extrapolating the dog predation events per day that tigers were monitored across a full year. This figure was also conservative, as locations were generally investigated as potential kill sites where tigers ceased moving for extensive periods of time (suggestive of feeding on a large animal) [33]. Tigers eating small animals like dogs are unlikely to remain long at such sites [34], and abandonment is likely, due to human disturbance. Dogs eaten per tiger in each time step were generated using a Poisson distribution based on the observed mean.

No data are available on the prevalence of CDV infection in dogs in the vicinity of SABZ, although serological surveys have detected antibodies in 58% of unvaccinated dogs in similar habitat and socio-economic conditions, indicating that infection is common [5]. CDV prevalence has been reported for dog communities in Thailand (2.93%) and South Africa (5.0%) [35], [36], with 1.5% dogs with respiratory disorders infected in Japan [37]. Alternate scenarios based on extremes of published data were used to estimate low risk (mean dog predation = 0.27 dogs/tiger/year, and dog prevalence = 1.5%) and high risk (mean dog predation = 1.66 dogs/tiger/year, and dog prevalence = 5.0%) of infection.

### 2.6. Wild carnivore to tiger infection

Two sources were used to estimate the rate at which tigers predate wild carnivores. The first used data on kills reported by forest guards from 1933 to 1994 [22]. Using a median kill frequency of 7.5 days [38], and the percentage of total kills that were carnivores, we estimated a figure of 1.65 wild carnivore kills/tiger/year. The second estimate was based on the radiotelemetry dataset described above for dogs, and generated an estimate of 3.87 wild carnivore kills/tiger/year. Tiger contact with other carnivores was limited to the period 7 April to 3 November to account conservatively for seasonal hibernation of several abundant prey species.

No data exist on the prevalence of CDV in wild carnivores in SABZ. Therefore, published data from other regions were used to estimate the range of CDV prevalence in SABZ. European sources reported CDV prevalence of 0.6% in mustelids in the Czech Republic [39], and 6.2% of foxes and mustelids in Germany [40]. An model of CDV outbreaks in Italian red foxes estimated a prevalence of <4% during epidemic peaks and a 2% prevalence in live foxes overall [41]. Risk scenarios were generated as for dog exposure, with low risk (mean predation = 1.65 carnivores/tiger/year; prevalence = 0.6%) and high risk (mean predation = 3.87 carnivores/tiger/year; prevalence = 6.2%) of infection.

### 2.7. Tiger to tiger infection

The model assumed that intraspecific interactions were limited to contact between tigers of opposite sexes. Females were assumed to interact with a male once per month, and males contacted multiple females at a rate of two interactions per month. These rates are conservative as they ignore the potential for distemper transmission via scent marks (which has yet to be evaluated), as well as between males encroaching on neighbouring territories. The probability of becoming infected during an interaction was based on the CDV prevalence *p* during the time step. Infected tigresses with dependent cubs were assumed to transmit the virus to their litter, with cubs not surviving their mother’s death.

### 2.8. Cycles of CDV infection

In some wildlife populations CDV is observed to occur in cycles of epizootic waves [42]. To simulate this, the model was run using a background of low infection risk, with epizootic years of high infection risk every three, five or seven years, to reflect reported periods of epizootic waves (File S1), as well as with control simulations using mean annual CDV prevalence across the varying cycles.

### 2.9. Simulating the effect of population size

To investigate how CDV infection influenced the 50-year extinction probability of populations with variable initial sizes, model simulations were run with and without CDV for founder populations between three and 288 tigers. A moderately low risk infection scenario was chosen for simulating CDV infection. A CDV prevalence of 1.5% was selected for domestic dogs, as this estimate reproduced an observed seroprevalence of 58% [5], and 2.0% was selected as a moderate non-outbreak value for wild carnivores, following the detailed treatment by Nouvellet et al. (2013). The rate of effective contact for both domestic dogs and wild carnivores was set to low risk, with 0.27 and 1.65 animals per tiger per year respectively. Tiger mortality from CDV was set to 40%. The model was allowed to equilibrate for 40 years, at which point CDV was introduced, then run for a further 50 years. A total of 1,000 simulations were run for each starting population; 50-year extinction probabilities were calculated as the proportion of simulations with extant populations in year 40 that subsequently reduced to zero.

### 2.10. Simulating alternative infection scenarios

Seventeen infection scenarios (Table 1) were simulated to determine the respective impact of potential modes of infection, duration of infectious period, mortality rate and cyclic period of epizootic waves. The impact of each scenario was determined using two output parameters, population growth rate (λ) and 50-year extinction probability.

**Table 1:** Summary of model scenarios.

		Dog		Wild carnivore							
Scenario number	Scenario name	Prevalence(%)	Mean effectivecontact	Prevalence (%)	Mean effective contact	Tiger-tiger Transmission	Infectious period (days)	Mortality (mean death per infection)	Population growth (λ)		Percentagepopulationsextinctin 50 years
1	Control	0	0	0	0	No	0	0	1		23.5%
2	Low risk dog	1.5	0.27	0	0	Yes	45	0.4	0.987		29.8%
3	High risk dog	5.0	1.66	0	0	Yes	45	0.4	0.958		66.1%
4	Low risk wildlife	0	0	0.6	1.65*	Yes	45	0.4	0.984		33.4%
5	High risk wildlife	0	0	6.2	3.87	Yes	45	0.4	0.956		74.5%
6	Low risk dog + wildlife	1.5	0.27	0.6	1.65*	Yes	45	0.4	0.980		35.7%
7	High risk dog + wildlife	5.0	1.66	6.2	3.87	Yes	45	0.4	0.955		79.3%
8	30 day infectious period	1.5	0.27	0.6	1.65*	Yes	30	0.4	0.995		30.3%
9	60 day infectious period	1.5	0.27	0.6	1.65*	Yes	60	0.4	0.966		40.7%
10	Low mortality	1.5	0.27	0.6	1.65*	Yes	45	0.3	0.982		34.0%
11	High mortality	1.5	0.27	0.6	1.65*	Yes	45	0.5	0.978		38.5%
12	3-year infection cycle	1.5 or 5.0	1.66	0.6 or 6.2	3.87	Yes	45	0.4	0.959		69.1%
13	5-year infection cycle	1.5 or 5.0	1.66	0.6 or 6.2	3.87	Yes	45	0.4	0.962		64.2%
14	7-year infection cycle	1.5 or 5.0	1.66	0.6 or 6.2	3.87	Yes	45	0.4	0.964		61.3%
15	Mean of 3 yr cycle	2.7	1.66	2.4	3.87	Yes	45	0.4	0.961		67.7%
16	Mean of 5 yr cycle	2.2	1.66	1.7	3.87	Yes	45	0.4	0.963		66.5%
17	Mean of 7 yr cycle	2.0	1.66	1.4	3.87	Yes	45	0.4	0.963		65.1%

Details of the fifteen canine distemper virus (CDV) infection scenarios used in the model simulations, used to determine tiger population growth rate (*lambda*, λ, calculated through 50 simulations with a founder population of 200 female and 100 male tigers) and 50-year extinction probability (calculated through 1,000 simulations as the proportion of simulations where population were reduced to zero before the run was complete).

*Derived from 3.4% of 551 kills per year comprising wild carnivore prey (30), and a median of one kill every 7.5 days (or 48.53 kills/year) (42).

To estimate λ the model was run with a population suitably large enough to enable determination of λ in the absence of extinction. A calculation of λ was made for the 50 years following CDV introduction at year 40, as the exponent of the coefficient from linear regression of log-transformed population size and year of simulation. Mean values of λ were calculated by repeating the simulations 50 times. Extinction probabilities were estimated as described above with a founder population to reflect maximum holding capacity for SABZ, as described earlier.

The performance of the model in the absence of CDV was verified through comparison with a Lefkovitch matrix, which gave similar estimates of λ to those produced by the full model.

## Results

**Figure 1:**
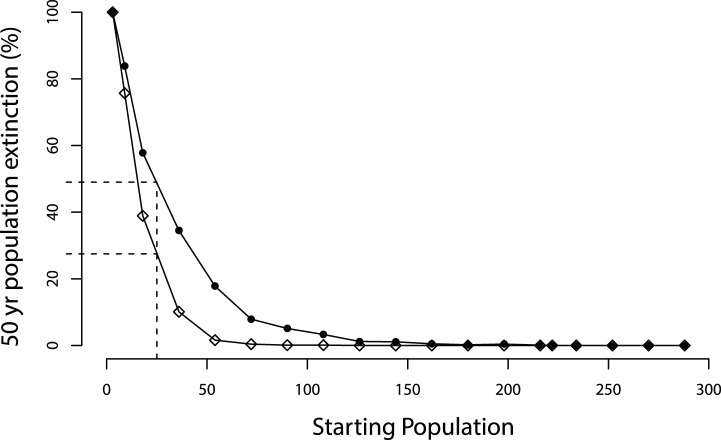
50-year extinction probabilities for tiger populations of variable size. Points illustrate the mean probability that a tiger population of given starting size will decline to extinction over 1,000 model simulations both with canine distemper virus (CDV) infection (black dots) and a control scenario without CDV (open diamonds).

As tiger founder population declined below a threshold, the 50-year extinction probability was observed to increase (Figure 1) both for populations exposed to CDV and controls. However, this threshold was much higher for CDV populations (founder population of 219), than controls (founder population of 108). Below these threshold values, a greater proportion of CDV populations declined to extinction than controls of equivalent founder size, converging at a founder population of three tigers.

**Figure 2:**
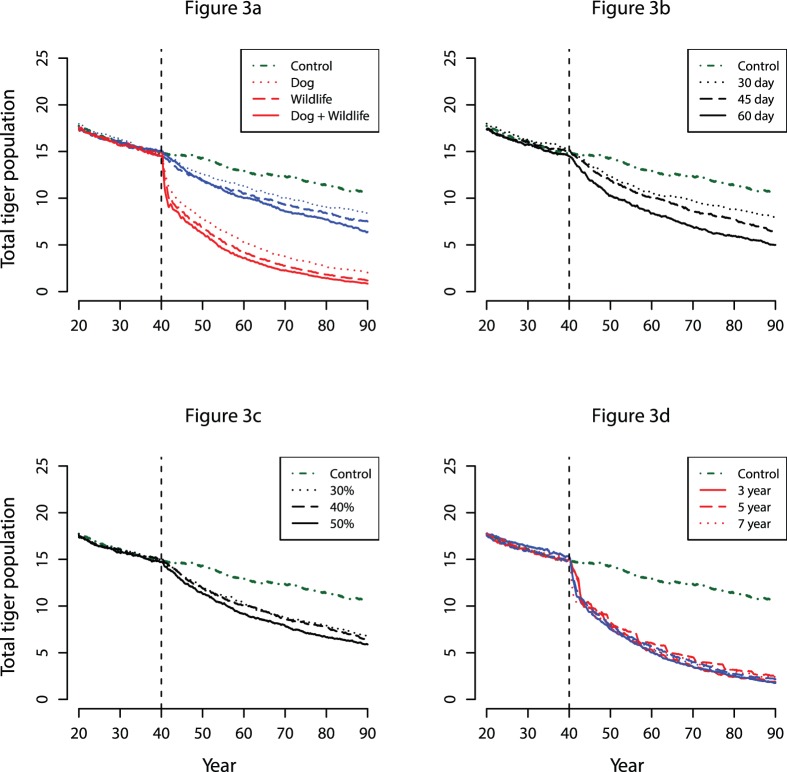
Mean population growth rate for canine distemper virus (CDV) infection scenarios in an Amur tiger population. Mean growth rate (λ) was obtained through fifty model simulations, using a variety of infection scenarios including a) direct transmission of CDV through tiger predation of dogs, wild carnivores (wildlife) and both dogs and wild carnivores combined at low and high risk infection rates, b) low risk infection through predation of dogs and wild carnivores and an infectious period of 30, 45 and 60 days, c) low risk infection through predation of dogs and wild carnivores with mortality rate of 30%, 40% and 50%, and d) enzootic infections cycles of three, five and seven years alongside equivalent mean values without cycling. Tiger-to-tiger infection is included in all infection scenarios. The dashed line indicates the threshold growth rate, below which populations will become extinct within one hundred years.

**Figure 3:**
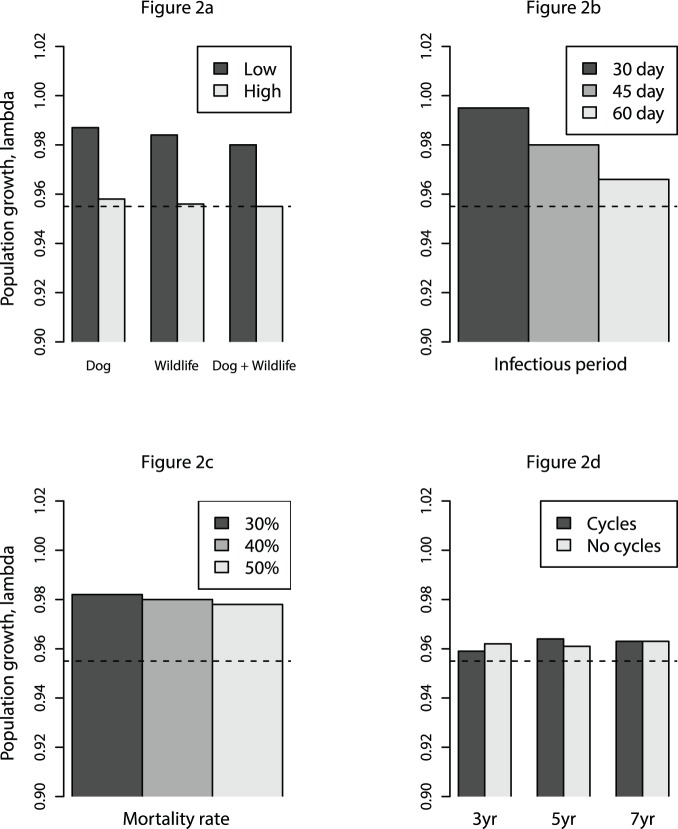
Changes in mean tiger population size over time under various canine distemper virus (CDV) infection scenarios. Mean tiger population size was obtained through one thousand model simulations. Starting populations of 17 territorial females and 5 territorial males were used to simulate the tiger population in the Sikhote-Alin Biosphere Zapovednik, and CDV was introduced in year 40 (vertical dashed line). Individual plots illustrate a) direct CDV infection between tigers and dogs, wild carnivores (both individually and combined), under low risk (blue) and high risk (red) infection scenarios, b) the effect of varying the duration of tiger infectious period at 30 days, 45 days and 60 days with tigers exposed to dogs and wild carnivores under conditions of low risk of infection, c) the effect of varying mortality rate of infected tigers carnivores under conditions of low risk of infection, and d) the effect of varying epizootic infection cycles of three years, five years and seven years with tigers exposed to dogs and wild carnivores under conditions of high risk of infection in epizootic years, and low risk in all other years (red), and holding prevalence at a constant equivalent to the mean annual prevalence of three, five and seven year infection cycles (blue). Control scenarios are represented in all panels (green). Tiger-to-tiger transmission is permitted in all model runs.

Low risk infection scenarios, with tigers exposed through contact with dogs, wild carnivores and a combination of the two (scenarios 2, 4 and 6) had a minor impact on population growth rate or extinction risk (Table 1; Figures 2a, 3a). When exposed to both infected dogs and wild carnivores at low infection risk, tiger population growth was reduced to 0.980 compared to the control value of 1.000, a decline of 64% if sustained over 50 years (Table 1; Figure 3a). Equivalent scenarios with a high risk of infection (scenarios 3, 5 and 7) resulted in greater extinction probabilities of 42.6%, 51.0% and 55.8% above that of the control population, respectively (Table 1; Figures 2a, 3a).

Variation in the duration of CDV infectious period in infected tigers (scenarios 6, 8 and 9) had a modest impact on λ (range 0.995 to 0.966), (Table 1; Figures 2b, 3b), indicating that tiger-tiger transmission influenced the outcome. The impact of variation in mortality rate (scenarios 6, 10 and 11) was approximately proportional to the impact on λ and population extinction (Table 1; Figures 2c, 3c). Population growth and extinction probability was the same whether CDV was introduced in a cyclical pattern, or remained identical in all years when mean annual prevalence was held constant (scenarios 10–15) (Table 1; Figures 2d, 3d).

## Discussion

Primary threats to global tiger conservation are increased adult mortalities from poaching and human conflict, reduction in available prey and loss of suitable habitat [1], [43]. Our findings suggest that a multi-host pathogen such as CDV may exert an additional negative influence on tiger population dynamics. Given that tigers now occupy <7% of their former range [43], and more than half of the world’s tigers now exist in populations of less than 25 individuals [44], any factor exacerbating extinction threats needs careful evaluation. The capability of CDV to infect multiple host species effectively removes the density-dependant effect that would regulate an endemic tiger pathogen. As tiger populations decrease, infection in the reservoir population remains unchanged, adding to the challenges of managing small and isolated populations in a landscape hostile to tigers [45]. Using a conservative CDV infection scenario, our simulation found that a population of 25 individuals was 1.65 times more likely to decline to extinction than a population affected by stochasticity alone.

While CDV infections have led to the extinction or near extinction of small, fragmented or depleted populations [10]–[12], other carnivore communities appear to tolerate exposure with negligible impacts on population viability. Models indicate that tigers are less resilient to increases in mortality than cougars (*Puma concolor*) and leopards (*Panthera pardus*), as they breed later and have longer inter-birth intervals [4]. Female cougars reach sexual maturity at 24 months and leopards at 36 months, and exhibit inter-birth intervals of 18 and 20 months respectively [4]. By comparison, female Amur tigers have their first litters at approximately 42–54 months, with a mean inter-birth interval of 22 months [24]. Tiger populations also take longer to recover from periods of increased adult mortality, and these reduced populations are more prone to extinction from environmental stochasticity or other challenges [3]. However, lion surveys in southern Africa have found no evidence for reduced survival or population declines despite widespread CDV exposure [46]. Other populations such as lions in East Africa have undergone dramatic population declines as a result of CDV outbreaks, while on other occasions the virus appears to have circulated as a ‘silent’ infection, with little apparent pathogenicity and no population impact [18]. This may hint at a more complex aetiology, with additional factors such as co-infections determining the magnitude of any population effects [18].

Despite these qualifications, and recognising that the population significance of CDV may be complex, it is important that these not be used as grounds for complacency. There have been many case reports involving CDV in captive tigers, which appear to have been uncomplicated by co-infection with other pathogens, and resulted in high rates of mortality despite supportive care [13], [14], [30], [47]. Lethal infections have also been reported in Russia in 2003 and 2010 [6], [7], and more recently in India, confirming the presence of infections within other wild populations as well [8]. While these could represent incidental cases, diagnosing infectious disease is very challenging in such cryptic and wide-ranging animals, and a large proportion of cases likely remain unidentified. Coincident with the two cases diagnosed in 2010, there was a sharp decline in the population of tigers in SABZ, from a peak of 38 individuals in 2007 to a low of nine in 2012. Although causality is difficult to confirm, there was no evidence of an increase in poaching in or around the reserve at this time. With the loss of several breeding age animals, the recovery of two carcasses of collared tigers with no signs of human interference, and observation of behaviour consistent with CDV in another undiagnosed collared tiger, it seems likely that CDV played at least a contributory role in the population decline.

The paucity of data on the status and epidemiology of CDV within the SABZ ecosystem limits the conclusions that can be drawn on the long-term viability of this particular population. Although uncertainties over some parameters, such as the duration of infectious periods and mortality rate, have relatively modest overall impact, the model is highly sensitive to variation in other parameters, particularly prevalence and effective contact rate. A broad range of population responses occurred when setting dog and wild carnivore prevalence and contact rates from low- to high-risk of infection, extending from negligible impact to 50-year extinction probability as much as 55.8% higher than populations without CDV. CDV prevalence estimates relied on a small number of published sources from other regions, and are the greatest source for uncertainty. Neither of the sources used as a basis for selecting wild carnivore prevalence levels for the low-risk (0.6%) or high-risk (6.2%) scenarios discussed the epidemiological context of their sample sets [39], [40]. Thus it was unclear whether these figures were derived during periods of CDV outbreaks, or represented background levels between outbreaks. The only study to address this used a modelling approach based on surveillance data for CDV in foxes in Italy, and estimated a CDV prevalence of 2.0% over a study period that spanned outbreak and non-outbreak periods [41]. This estimate fell between our low and high-risk scenarios, but in lying below the median value suggests that our high-risk estimate may have been unrealistically high.

One important aspect to consider when applying the model findings to the SABZ study is the omission of tiger migration into, or dispersal from the study area, which is an important feature of this population, as the Zapovednik lies within an extensive matrix of suitable tiger habitat [48]. Historically tigers were eradicated from this area, but began to recolonize in the 1960s as the result of increased protection and immigration from surrounding areas [32]. More recently, SABZ has acted as a “Source Site”, with tigers born in the Zapovednik dispersing beyond reserve boundaries to other suitable habitat [1]. Such movements would help buffer against declines that were confined to a limited geographical area. However, CDV may require reservoirs that occupy wide areas in order to persist [49], and the observation of two tiger cases in 2010 that were over 300 km apart suggests that the impacts of CDV may not be local in scale [7]. Many other tiger populations beyond Russia are more isolated and the prospect for migration is considerably lower [1]. The buffering effects of tiger immigration will not protect these sites, and the model may provide a more realistic assessment of their extinction probability.

Priorities for future research include the collection of field data to assess the prevalence of CDV shedders within populations of domestic and wild carnivores in SABZ and elsewhere. The data should assess temporal variation, which is evident in other species [42]. Determining the presence and periodicity of inter and/or intra-annual epizootic cycles, either through consultation with local veterinary authorities, or longitudinal, age-specific serosurveys, would elucidate how CDV prevalence varies over time. Studies should also focus on a broader range of multi-host pathogens, particularly rabies and bovine tuberculosis, as predator-prey interactions across a wider community of species could represent alternative means of exposing tigers to infectious diseases. Our findings indicate that the threat posed by multi-host disease should be considered wherever tigers coexist and interact with other carnivore species.

Importantly, this study supports conservation strategies based on securing large and inter-connected populations of tigers to ensure their long-term survival. The additive mortality arising from poaching, retaliatory killing and dog-transmitted diseases are a reflection of anthropogenic ‘edge effects’ that occur in fragmented habitats and are likely to threaten tiger populations across their range. In lieu of a practical means of delivering CDV vaccines to wild tigers, the most viable strategy to ensure their conservation is the maintenance of large connected populations within protected areas that buffer the effects of local declines.

The densities of tigers, humans and dogs in the Russian Far East are some of the lowest in tiger range, and the tiger population is one of the largest and most widely dispersed throughout an extensive contiguous habitat. Most other tiger populations are restricted to small islands surrounded by human populations as dense as 1000/km^2^, with tiger density as high as 15–20/100 km^2^. In these situations there is likely to be much greater rates of tiger-dog, tiger-tiger, tiger-other carnivore interactions and hence potentially much greater risk of CDV transmission. Although the distinct climatic conditions in Russia may enhance winter transmission of CDV, our model took a conservative approach and ignored the potential for the virus to remain viable in the environment. Our findings therefore have important implications for tigers in other range states, highlighting a need to assess the reservoir dynamics of CDV in these distinct ecosystems to better assess the conservation threats to remaining tiger populations.

## Supporting Information

File S1 **A summary of sources used for parameterizing the model is available online.**
(DOCX)
