# Supplementary material for: Estimating the Potential Impact of Canine Distemper Virus on the Amur Tiger Population (Panthera tigris altaica) in Russia
Source: PLoS One. 2014 Oct 29;9(10):e110811. doi: 10.1371/journal.pone.0110811 (PMC4212977; doi:10.1371/journal.pone.0110811)
Supplement: File S1 — A summary of sources used for parameterizing the model is available online. (DOCX) [file pone.0110811.s001.docx]

**File S1.** Model parameters

**A. Survival**

Source: [1]

- Annual survival rate of adult (>36 mo) females (0.81 +/-0.10)
- Annual survival rate of subadult (18-36 mo) females (0.72+/-0.25)
- Annual survival rate of adult (>36 mo) males (0.63+/-0.20)
- Annual survival rate of subadult (18-36 mo) males (0.41+/-0.46)

Source: [2]

- Reproductive rate was 1.4 cubs female^-1^, but only half of these (0.7 cubs/year) survived up to 12 months of age.
- Cub mortality was 41– 47% during the period from 1st detection to 12 months of age, the range depending on our methods (but note that actual mortality in first year will be higher due to mortality prior to first observation).

*Interpretation:*

Based on this, reasonable estimates might be:

| Tiger age class | Equivalent age (mo) | Annual survival (female) | Annual survival (male) |
| --- | --- | --- | --- |
| 1 | 0-12 | 0.50 | 0.50 |
| 2 | 12-24 | 0.61 | 0.46 |
| 3 | 24-36 | 0.72 | 0.41 |
| 4 | 36-48 | 0.81 | 0.63 |
| 5 | >48 | 0.81 | 0.63 |

**B. Reproduction**

Source: [2]

- Minimum age of 1st reproduction as 4 +/-0.4 years (95% CL)
- Gestation between 101 and 108 days
- Females produce 1-4 cubs (mean: 2.4; SD: 1.15) – Note cannot account for pre-observation losses, so numbers at birth likely to be higher.
- Mean age at dispersal was 18.8 +/-1.5 months (n =5).
- Reproductive rate was 1.4 cubs female^-1^, but only half of these (0.7 cubs/year) survived up to 12 months of age.

**C. Exposure to infected dogs**

*a) Contact rate*

Source: [3]

| Year | Females | Males | Subadults | Total tigers | Dog eaten | Dogs/  Tiger |
| --- | --- | --- | --- | --- | --- | --- |
| 1983 | 5.5 | 3 | 3 | 11.5 | 2 | 0.174 |
| 1984 | 8 | 3.5 | 5.5 | 17 | 5 | 0.294 |
| 1985 | 8 | 3.5 | 5.5 | 17 | 9 | 0.529 |
| 1986 | 7 | 3.5 | 5.5 | 16 | 19 | 1.188 |
| 1987 | 7 | 4.5 | 5 | 16.5 | 3 | 0.182 |
| 1988 | 8 | 3.5 | 9 | 20.5 | 2 | 0.098 |
| 1989 | 8 | 4.5 | 5.5 | 18 | 1 | 0.056 |
| 1990 | 9 | 3.5 | 4.5 | 17 | 2 | 0.118 |
| 1991 | 8 | 3.5 | 4.5 | 16 | 2 | 0.125 |
| 1992 | 7 | 4 | 10 | 21 | 2 | 0.095 |
| 1993 | 8.5 | 4 | 5.5 | 18 | 2 | 0.111 |

Mean dogs eaten per tiger/year = 0.27

Source: [4]

| Tiger ID | Sex | Days monitored | Domestic dog | Total domestic dogs |
| --- | --- | --- | --- | --- |
| Pt99 | F | 481 | 3 | 3 |
| Pt90 | M | 45 | 0 | 0 |
| Pt100 | M | 99 | 0 | 0 |
| Pt97 | F | 36 | 0 | 0 |
|  |  | **Total:** 661 |  | **Total:** 3 |

Mean dogs eaten per tiger/year = 1.66

*b) CDV prevalence*

Source: [5]

RT-PCR data from Bangkok found CDV in 2.94% of healthy dogs (n=102), and 2.75% of dogs with respiratory signs (n=109).

Source: [6]

RT-PCR data from Japan found CDV in 1.5% of dogs with respiratory signs (n=68)

Source: [7]

Study of domestic dogs in a rural South African town (n=220) found that 5% were positive for CDV.

**D. Exposure to infected wild carnivores**

*a) Contact rate*

Source: [4]

| Tiger ID | Sex | Days monitored | Black bear | Brown bear | Badger | Total wild carnivore |
| --- | --- | --- | --- | --- | --- | --- |
| Pt99 | F | 481 | 1 | 1 | 4 | 6 |
| Pt90 | M | 45 | 0 | 1* | 0 | 1 |
| Pt100 | M | 99 | 0 | 0 | 0 | 0 |
| Pt97 | F | 36 | 0 | 0 | 0 | 0 |
|  |  | Total: 661 |  |  |  | Total: 7 |

* Predation not confirmed, but certainly interaction

Mean wild carnivores eaten per tiger/year = 3.87

Source: [8]

3.4% of 552 kills comprised carnivore species.

*b) CDV prevalence*

Source: [9]

Screened wild mustelids, including 173 from trappers and one (0.6%) was positive for CDV

Source: [10]

RT-PCR used to screen foxes (predominantly) and mustelids from Germany found 6.2% positive for CDV (n=275). No comment made regarding whether this was during epizootic.

Source: [11]

Antigen demonstrated in 37% (n=236) wild carnivores examined. However, animals were collected as suspect rabies cases, so clear bias.

*c) Epizootiology*

Source: [12]

Discusses cyclical waves of CDV in raccoons in New Jersey with 4 year period.

Source: [13]

Discusses 3-7 year waves in prairie ecosystems.

Source: [14]

Anecdotal comment that CDV epizootics occur every 5 years in Nairobi and Mombasa.

**E. Infection**

*a) Outcome of infection*

Captive papers are pretty inconsistent in reporting. Generally focus on animals succumbing to disease, with progressively less attention to recovered animals or exposed animals.

| Animal name/origin  (species) | Clinical cases | | Exposed | Period of clinical signs | Source |
| --- | --- | --- | --- | --- | --- |
|  | Died | Recovered |  |  |  |
| 6 yr male  (tiger) | 1 | 0 | ? | Presented with CNS signs died after 2 months | [15] |
| Blythe et al. | 2 | 0 | 2? | One animal died as a cub after 1 wk with GIT signs. The other recovered, but after 5 months developed CNS signs, which continued until euthanasia at 18 mo. | [16] |
| Shambala (tigers) | 2 | 4 | ? | Unknown | [17] |
| Wildlife Waystation (tigers) | 4 | ? | ? | 4 cats found moribund with no signs. 29 cats GIT/Resp signs for 1-2 weeks (14 cats recovered with supportive care). 15 cats then developed CNS signs (plus 2 spontaneous cases), lasting from 1-2d (the majority), up to 2wks. | [17] |
| Wildlife Waystation (big cats) | 17 | 18 | ? (39 clinically unaffected) | (Discussed above) | [17] |
| Zenker et al. (tigers) | 5 | 2 | ? | “several weeks to months” | [18] |
| Morka  (tigers) | 1 | 0 | 1 | Showing clinical signs at presentation, died 44 days later. | [19] |
| Zagreb (tigers) | 2 | 0 | 2? | 2-4 days | [20] |
| Nagao et al.  (tigers) | 3 | 9 | 22? (10 untested and clinically unaffected) | Onset GIT/Resp signs in late January, two deaths (Jan 21, 5 Feb). one cat developed CNS signs and died (11 Mar). | [21] |

**References**

1. Goodrich JM, Kerley LL, Smirnov EN, Miquelle DG, McDonald L, et al. (2008) Survival rates and causes of mortality of Amur tigers on and near the Sikhote-Alin Biosphere Zapovednik. J Zool 276: 323–329. doi:10.1111/j.1469-7998.2008.00458.x.

2. Kerley LL, Goodrich JM, Miquelle DG, Smirnov EN, Quigley HB, et al. (2003) Reproductive parameters of wild female Amur (Siberian) tigers (Panthera tigris altaica). J Mammal 84: 288–298.

3. Smirnov EN, Miquelle DG (1999) Population dynamics of the Amur tiger in Sikhote-Alin Zapovednik, Russia. In: Seidensicker J, Christie S, Jackson P, editors. Riding the tiger; tiger conservation in human-dominated landscapes. Cambridge, UK. pp. 61–70.

4. Miller CS (2012) Amur tiger predation and energetic requirements in the Russian Far East: New insights from global positioning collars University of Montana.

5. Posuwan N, Payungporn S, Thontiravong A, Kitikoon P (2010) Prevalence of respiratory viruses isolated from dogs in Thailand during 2008-2009. Asian Biomed 4: 563–569.

6. Mochizuki M, Yachi A, Ohshima T, Ohuchi A, Ishida T (2008) Etiologic Study of Upper Respiratory Infections of Household Dogs. J Vet Med Sci 70: 563–569.

7. Rautenbach G, Boomker J, de Villiers I (1991) A descriptive study of the canine population in a rural town in southern Africa. J S Afr Vet Assoc 62: 158–162.

8. Miquelle DG, Smirnov EN, Quigley HG, Hornocker MG, Nikolaev IG, et al. (1996) Food habits of Amur tigers in the Sikhote-Alin Zapovednik and the Russian Far East, and implications for conservation. J Wildl Res 1: 138–147.

9. Pavlacik L, Celer V, Koubek P, Literak I (2007) Prevalence of canine distemper virus in wild mustelids in the Czech Republic and a case of canine distemper in young stone martens. Vet Med (Praha) 52: 69–73.

10. Frolich K, Czupalla O, Haas L, Hentschke J, Dedek J, et al. (2000) Epizootiological investigations of canine distemper virus in free-ranging carnivores from Germany. Vet Microbiol 74: 283–292. doi:10.1016/S0378-1135(00)00192-9.

11. Van Moll P, Alldinger S, Baumgärtner W, Adami M (1995) Distemper in wild carnivores: an epidemiological, histological and immunocytochemical study. Vet Microbiol 44: 193–199.

12. Roscoe E (1993) Epizootiology of canine distemper in New Jersey raccoons. J Wildl Dis 29: 390–395.

13. Williams ES (2001) Canine Distemper. In: Williams ES, Barker IK, editors. Infectious Diseases of Wild Mammals Third Edition. Ames, Iowa: Iowa State University Press. pp. 50–59.

14. Alexander KA, Appel MJG (1994) African wild dogs (Lycaon pictus) endangered by a canine distemper epizootic among domestic dogs near the Masai Mara National Reserve, Kenya. J Wildl Dis 30: 481–485.

15. Gould DH, Fenner WR (1983) Paramyxovirus-like nucleocapsids associated with encephalitis in a captive Siberian tiger. J Am Vet Med Assoc 183: 1319–1322.

16. Blythe LL, Schmitz JA, Roelke M, Skinner S (1983) Chronic encephalomyelitis caused by canine-distemper virus in Bengal tiger. J Am Vet Med Assoc 183: 1159–1162.

17. Appel M, Yates R, Foley G, Bernstein J, Santinelli S, et al. (1994) Canine distemper epizootic in lions, tigers, and leopards in North America. J Vet Diagnostic Investig 6: 277–288.

18. Zenker W, Koelbl S, Reifinger M, Kuebber-Heiss A, Airikkala I, et al. (2001) A possible case of distemper in Siberian tigers. Erkrankungen der Zootiere 40: 221–228.

19. Quigley KS, Evermann JF, Leathers CW, Armstrong DL, Goodrich J, et al. (2010) Morbillivirus Infection in a Wild Siberian Tiger in the Russian Far East. J Wildl Dis 46: 1252–1256.

20. Konjevic D, Sabocanec R, Grabarevic Z, Zurbriggen A, Bata I, et al. (2011) Canine distemper in Siberian tiger cubs from Zagreb ZOO: case report. Acta Vet Brno 80: 47–50.

21. Nagao Y, Nishio Y, Shimoda H, Tamaru S, Shimojima M, et al. (2012) An Outbreak of Canine Distemper virus in Tigers (Panthera tigris): Possible Transmission from Wild Animals to Zoo Animals. J Vet Med Sci 74: 699–705.
